# Postoperative foreign body retention following anal injury leading to a giant bladder stone: a case report

**DOI:** 10.1186/s12894-026-02210-5

**Published:** 2026-06-02

**Authors:** Shuhong Wang, Yin Tian, Xiangui Zhou, Peng Wang, Peng Ye, Yuanlan Lu, Anyong Yu

**Affiliations:** 1 Department of Emergency, Affiliated Hospital of Zunyi Medical University, Zunyi, Guizhou 563000 China; 2 Department of Cardiology, The First People’s Hospital of Zunyi, Zunyi, Guizhou 563000 China

**Keywords:** Fall from height, Anal trauma, Foreign body retention, Giant bladder stone, Adolescent

## Abstract

**Purpose:**

Penetrating anal injuries resulting from falls from height are a rare form of high-energy trauma, often accompanied by multiple organ injuries and delayed complications; giant bladder stones caused by retained wooden foreign bodies are particularly rare and highly prone to misdiagnosis. This study reports a case of this rare condition and highlights the key factors for achieving the best possible outcome.

**Methods:**

This study presents the case of a 14-year-old male patient who sustained multiple penetrating anal injuries caused by a wooden foreign body following a fall from a height. Abdominal CT was used to assess the location of the foreign body and the extent of organ injury; the patient underwent emergency open abdominal surgery and transcatheter arterial embolisation for haemostasis, followed by long-term follow-up involving further imaging and interventional procedures as new symptoms arose.

**Results:**

Initial abdominal CT revealed a foreign body in the abdomen and pelvis, accompanied by ileal and bladder injuries. Gross hematuria was present at admission, and urinary catheterization was maintained for 14 days postoperatively. The patient was discharged on postoperative day 20. Following emergency surgery and embolisation, the patient was discharged on the 20th postoperative day. Eight and a half years later, the patient developed lower urinary tract symptoms; a urinary tract CT scan revealed a giant bladder stone surrounding the residual foreign body, and the patient made a full recovery following surgical removal.

**Conclusion:**

For such patients, early and accurate diagnosis, prompt emergency surgery, thorough intraoperative exploration, objective assessment of bladder healing, and continuous long-term follow-up are crucial for preventing late complications and achieving the best possible outcomes.

**Supplementary Information:**

The online version contains supplementary material available at 10.1186/s12894-026-02210-5.

## Introduction

Anal foreign body injuries are a rare form of acute traumatic injury in clinical practice, occurring primarily in young adults [1]. The incidence of penetrating anal injury caused by a fall from height is extremely low, accounting for less than 1% of all blunt and penetrating abdominal traumas, and cases complicated by giant bladder stones due to retained wooden foreign bodies are even rarer, with only a few individual cases reported worldwide [1]. From an aetiological perspective, such injuries can be classified into two categories: intentional insertion and accidental trauma. Among these, penetrating anal injuries resulting from falls from a height constitute a rare form of high-energy trauma, often accompanied by concomitant injuries to the intestinal tract, urinary tract, blood vessels and retroperitoneal structures. Such injuries are characterised by acute onset, life-threatening nature, and significant diagnostic and therapeutic challenges. The development of giant bladder stones secondary to residual foreign bodies post-surgery is extremely rare, particularly in adolescent patients with multiple organ involvement [2]. Due to their low density and similarity to surrounding soft tissues, wooden foreign bodies are difficult to detect on imaging studies, making them prone to retention and the subsequent development of long-term complications [3, 4]. This paper reports a case of penetrating anal injury resulting from a fall from a height, which led to multiple organ injuries and was complicated by the long-term retention of a wooden foreign body, ultimately resulting in the formation of a giant bladder stone. We systematically review the clinical presentation, imaging characteristics, surgical management and key points of long-term follow-up in this case, with the aim of providing guidance for the diagnosis and treatment of similar cases.

## Case presentation

A 14-year-old male patient was admitted on 21 November 2015 following a fall from a height, during which a foreign object penetrated his anus, causing abdominal pain and bleeding for five hours.

**Fig. 1:**
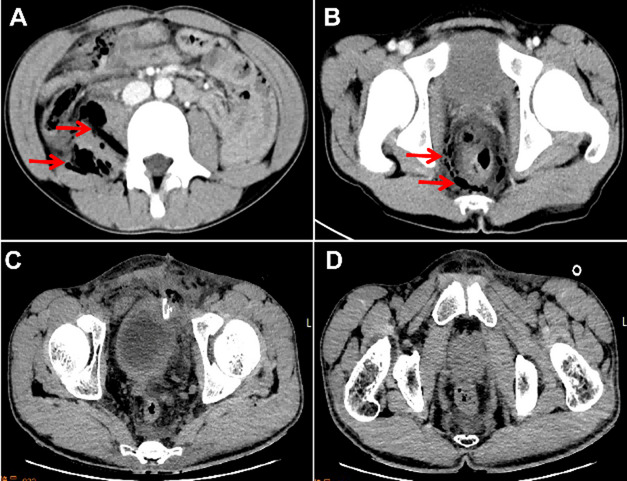
**A** and (**B**): Non-contrast CT scan of the abdomen, contrast-enhanced scan and 3D reconstruction performed at the time of injury. Multiple tubular and linear hypodense foreign bodies are visible in the mid-lower abdomen, right iliac region and pelvis; free air is present in the perianal region, pelvis and retroperitoneal space, accompanied by mild intra-abdominal haemorrhage and fluid collection, consistent with injury to the bowel and perianal soft tissues. **C** and (**D**): Follow-up abdominal CT scan on the second post-operative day, showing that the original intra-abdominal foreign bodies have been removed

Physical examination: Temperature 38 °C, pulse 110 beats per minute, respiratory rate 21 breaths per minute, blood pressure 135/77 mmHg. The patient is alert and oriented. There is widespread abdominal tenderness, rebound tenderness and abdominal muscle rigidity, with symptoms most pronounced in the lower abdomen; bowel sounds are not audible. A small amount of pale red bloody discharge is visible at the anus, accompanied by a mild perianal fissure. Digital rectal examination reveals a foreign body at the anus, with the examination glove stained with blood. Shortly after admission, gross haematuria was observed in the urine collected via the urinary catheter.Non-contrast, contrast-enhanced and 3D-reconstructed CT scans of the entire abdomen revealed multiple tubular and linear hypodense masses in the mid-lower abdomen, right iliac region and pelvis. Free gas was observed in the perianal region, pelvis and retroperitoneal space, accompanied by mild intra-pelvic haemorrhage and effusion, consistent with injury to the intestinal tract and perianal soft tissues.(Fig. 1A, B).

The preliminary diagnosis was open abdominal trauma (suspected injury to hollow viscera and the urinary system), diffuse peritonitis, intra-abdominal foreign bodies, and a laceration of the perianal skin. Preoperative preparations were initiated immediately upon admission, and an emergency laparotomy was performed, followed by resection of the lacerated ileum, intestinal anastomosis, and removal of the intra-abdominal foreign bodies. Intraoperative findings: A 10-centimetre-long laceration of the ileum was identified approximately 100 centimetres proximal to the ileocecal junction, accompanied by mucosal rupture and eversion. A wooden splinter extending to the posterior aspect of the right kidney was retrieved from the area anterior to the sacroiliac joint; two wooden foreign bodies, each 10 cm in length, were removed from the right colonic sulcus. An irregular laceration measuring 3 cm in length was noted on the posterior wall of the bladder. Intraoperative assessment of the bladder: examination through the 3 cm laceration and gentle palpation of the visible inner mucosa of the bladder revealed no obvious foreign bodies. A saline bladder lavage was performed; no wood debris was observed in the lavage fluid. The failure to detect this 10 cm × 2 cm foreign body constituted a critical oversight in this procedure: the fragment was embedded within the submucosal layer of the posterior bladder wall and the surrounding extravesical tissue, rendering it inaccessible or unobservable through the limited laceration, whilst its radiolucent nature further complicated identification. The torn ileum was resected and an end-to-end anastomosis was performed. The bladder laceration was repaired using two layers of sutures, and all visible foreign bodies were removed. A urinary catheter was inserted to provide continuous drainage.

The patient was transferred to the intensive care unit following surgery. A follow-up abdominal CT scan performed on the second postoperative day revealed damage to the right iliopsoas and quadratus lumborum muscles, suspected injury to the middle and lower segments of the intestine, and damage to the anus and surrounding soft tissues; the foreign body had been removed (Fig. 1C, D). On the fourth postoperative day, the patient suddenly passed 600 ml of bloody stool, accompanied by abdominal distension and pain. Blood pressure dropped to 110/65 mmHg, and heart rate rose to 125 beats per minute. Arterial haemorrhage was suspected. An emergency percutaneous femoral artery puncture was immediately performed, followed by angiography of the iliac and superior mesenteric arteries, which revealed a ruptured pseudoaneurysm at the bifurcation of the left internal iliac artery. Superselective coil embolisation was subsequently performed, and the bleeding was rapidly controlled. Bladder management: A urinary catheter was left in place for 14 days. Gross haematuria resolved on the 10th postoperative day, and urine culture results were negative. A cystogram was not performed prior to catheter removal to confirm bladder healing; this oversight resulted in the failure to detect the retained foreign body. The patient was discharged on the 20th postoperative day.

**Fig. 2:**
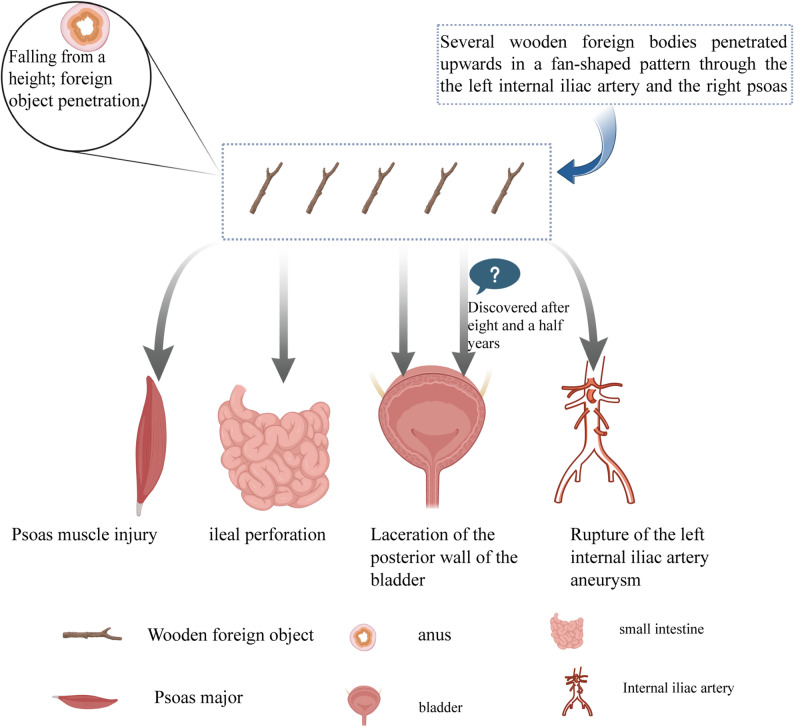
Schematic diagram of the fan-like penetration trajectories of multiple wooden foreign bodies from the anus, involving intraperitoneal (ileum) and extraperitoneal (bladder, left internal iliac artery, right psoas major) structures

Trajectory of injury: Multiple wooden foreign bodies penetrated through the anus, distributed in a fan-shaped pattern, causing injury to intra-abdominal (ileum) and extra-abdominal (bladder, left internal iliac artery, right psoas major muscle) structures (Fig. 2).

**Fig. 3:**
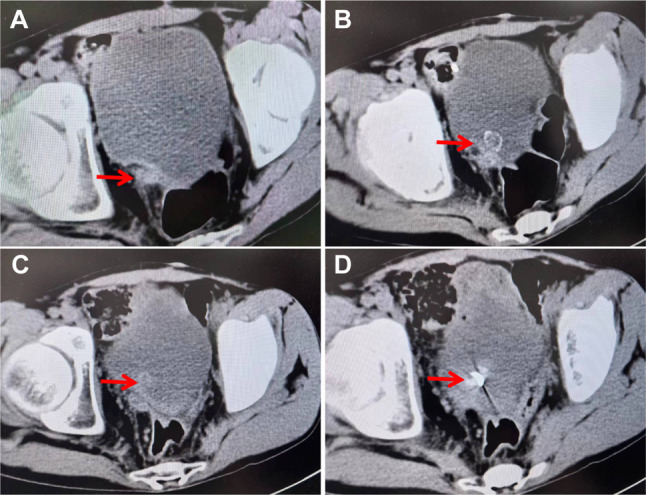
**A** and (**B**): A follow-up abdominal CT scan six months after the operation revealed residual embolisation coils in the right posterior pelvic cavity; no other abnormalities were observed. **C** and (**D**): A CT scan of the abdomen performed one year after surgery revealed a soft tissue mass in the right iliac fossa, consistent with the characteristics of a chronic infection and post-interventional complications

**Fig. 4:**
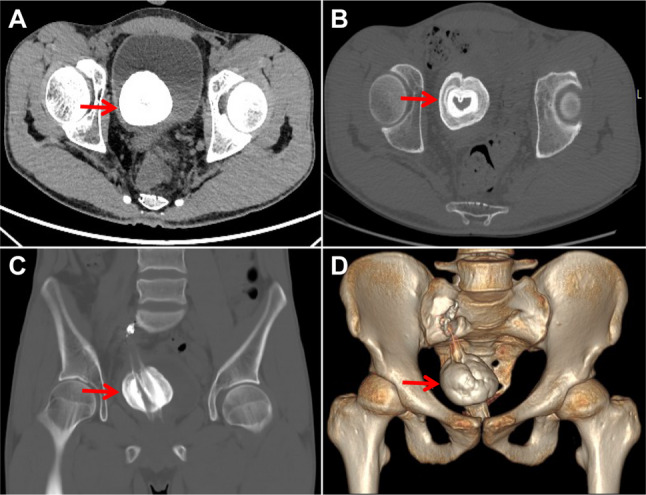
An 8½-year post-operative CT scan of the urinary system revealed an irregular, low-density mass within the bladder, consistent with a large stone (red arrow), accompanied by thickening of the posterior bladder wall, compression of the right ureteral orifice by the stone, and mild bilateral hydronephrosis. The panels show different views of the stone: **A** axial contrast-enhanced CT, (**B**) bone window axial CT, (**C**) coronal reformatted CT, and (**D**) three-dimensional volume-rendered reconstruction.

**Fig. 5:**
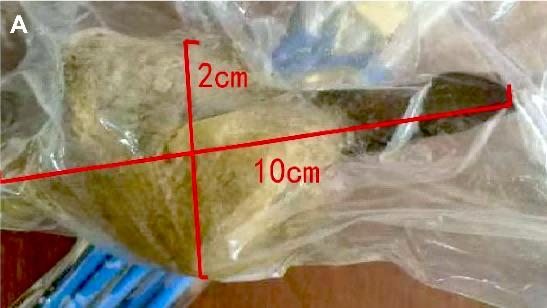
The foreign body and calculus removed during surgery consisted of a tree-like wooden object measuring approximately 10 cm × 2 cm, to which a massive calculus with a diameter of approximately 5 cm was attached

**Fig. 6:**
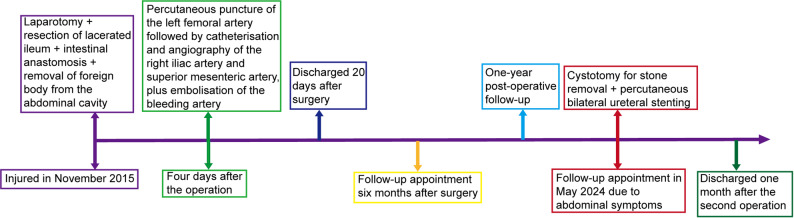
Clinical timeline illustrating the 8-year course of a 14-year-old male patient with a penetrating perineal injury from a fall from height. Initial management included emergency laparotomy with resection of lacerated ileum, anastomosis, and removal of abdominal foreign bodies, followed by transcatheter arterial embolization for postoperative hemorrhage. The patient was discharged 20 days after surgery with uneventful routine follow-up. In May 2024, the patient presented with abdominal symptoms, and imaging revealed a giant bladder stone secondary to residual wooden foreign body. Cystotomy for stone removal with bilateral ureteral stenting was performed, and the patient achieved full recovery and was discharged one month postoperatively

Routine follow-up CT at 6 months and 1 year showed embolisation coils and chronic inflammatory changes without symptoms (Fig. 3).Follow-up was interrupted for 7.5 years due to the patient’s loss to follow-up; during this period, no urine analysis, cystoscopy or urological imaging was performed.Eight and a half years later, he presented with frequency, urgency, and dysuria. Urinary CT demonstrated a giant bladder stone with bladder wall thickening and mild bilateral hydronephrosis (Fig. 4). Open cystolithotomy was performed, removing a 10 cm × 2 cm wooden foreign body with a 5 cm calculus attached (Fig. 5). The patient recovered uneventfully and was asymptomatic at 6-month follow-up (Fig. 6).

## Discussion

Anal penetration by a foreign body is a clinically rare form of acute trauma, primarily classified into two categories: accidental injury and self-insertion [1]. Among these, anal penetration caused by a fall from a height constitutes a rare form of high-energy trauma, most commonly seen in adolescents and young adults. It typically occurs when a patient lands on their buttocks during a fall, causing a foreign body to penetrate through the anus into the pelvic and abdominal cavities. This frequently results in multi-organ injury, including damage to the rectum, bladder and blood vessels. The condition is life-threatening, and delayed diagnosis and treatment can easily lead to fatal complications such as diffuse peritonitis and haemorrhagic shock. Currently, the majority of clinically reported cases of anal foreign body penetration involve adults who have self-inserted a single foreign body, resulting in injury to a single organ; cases of bladder stones secondary to long-term residual foreign bodies following surgery are extremely rare [5]. There are no clear reports indicating that adolescents have developed such complex complications as a result of high-energy trauma.

This report describes a 14-year-old adolescent patient who sustained multiple wooden foreign bodies penetrating the anus following a fall from a height, affecting the ileum, bladder, right psoas major muscle and internal iliac artery, resulting in multi-organ injury. During the acute phase, the patient developed complications including diffuse peritonitis and haemoperitoneum. In the early postoperative period, bleeding occurred due to the rupture of a pseudoaneurysm of the internal iliac artery; haemorrhage was controlled following percutaneous angiography and coil embolisation. During long-term postoperative follow-up (8.5years), residual foreign bodies were found within the bladder, leading to the formation of a giant calculus. Given the high diagnostic and therapeutic complexity and the unique nature of this case, it holds significant clinical reference value. The distinctiveness of this case is primarily reflected in three aspects: firstly, the unique mechanism of injury, involving the simultaneous penetration of multiple wooden foreign bodies, resulting in complex multi-system injuries; Secondly, complications of this condition are relatively rare and manifest in multiple stages, with early-stage arterial haemorrhage (associated with a high mortality rate) coexisting with late-stage secondary giant calculi; furthermore, there have been no previous reports of adolescent patients. Thirdly, the patient’s age presents particular challenges; at just 14 years of age, the patient is in a critical stage of growth and development, and organ function has not yet fully matured. Treatment must strike a balance between controlling the disease and preserving organ function, which represents a significant departure from the treatment priorities for adult patients.

The key to diagnosing this type of injury lies in rapidly determining the location of the foreign body, the extent of the injury and the organs involved. A detailed medical history and physical examination form the basis of the diagnosis; a preliminary diagnosis can be made by considering a history of a fall from a height, signs of peritonitis and a gentle digital rectal examination (to avoid displacing the foreign body); Contrast-enhanced abdominal CT scans and three-dimensional reconstruction are key diagnostic tools, as they clearly reveal the number and location of foreign bodies, as well as details of organ injury, providing precise guidance for surgery; if diagnostic paracentesis reveals non-clotting blood, this confirms the presence of intra-abdominal haemorrhage, which is a major indication for emergency surgery [3]. Where urinary tract injury is suspected, further investigations are required to confirm the diagnosis [3, 4]. The core principles of treatment include emergency surgery, foreign body removal, organ repair, infection control and prevention of complications, necessitating a staged, individualised treatment plan. During the acute phase, emergency laparotomy is performed to remove the foreign body, repair the ileum and bladder, control peritonitis, and identify occult vascular and bladder injuries. Bladder exploration must be thorough; For posterior wall lacerations, it is recommended to extend the scope of exploration or utilise cystoscopy to prevent foreign bodies from becoming embedded in the submucosal layer; the bladder must be thoroughly irrigated to detect minute debris. In the early postoperative period, interventional embolisation is used to treat arterial bleeding; this method is minimally invasive and provides reliable haemostasis, and has demonstrated its clinical value. In the long-term postoperative phase, residual foreign bodies may lead to secondary stone formation, which is associated with the friability of wooden foreign bodies, their deep location, difficulties in imaging detection, and postoperative infection [2]. Cystotomy is required to remove stones and foreign bodies, thereby relieving urinary tract obstruction and preserving renal function [5].

It should be noted that in cases of severe bladder trauma, objective confirmation via cystography or cystoscopy must be obtained prior to catheter removal; reliance on clinical symptoms alone is insufficient. The failure to perform cystography in this case was a critical oversight that led to the residual foreign body not being detected in a timely manner. The most common cause of anal penetration in adults is self-inflicted injury; however, cases in adolescents where multiple foreign bodies become embedded in the body following a fall from a height, resulting in multi-organ injury, are relatively rare. Residual wooden foreign bodies are difficult to identify on imaging studies [1, 6]. Furthermore, the patient in this case underwent interventional embolisation following surgery; the residual coils interfered with subsequent imaging follow-up, making the condition more prone to being missed and increasing the risk of secondary stone formation and infection. Although interventional embolisation is highly effective, long-term follow-up is still required to monitor vascular recovery. For complex pelvic and urinary tract trauma, follow-up should not be limited to one year. It is recommended that routine urine analysis be performed every 6–12 months to detect microscopic haematuria or pyuria, and that periodic cystoscopy be carried out every 1–2 years to allow for direct visual inspection, thereby monitoring for transmittable foreign bodies. The interruption in follow-up for a period of up to 7.5 years directly led to the delayed diagnosis of a giant bladder stone.

Based on the clinical management experience of this case, the following conclusions are drawn: in the emergency department, rapid assessment is crucial; a CT scan should be performed as early as possible to clarify the condition and avoid treatment delays; surgical exploration must be thorough to strictly prevent residual foreign bodies and occult injuries; close postoperative monitoring is required; when residual foreign bodies are suspected, a multi-pronged approach should be adopted, and early complications should be addressed promptly to reduce mortality [5].Long-term follow-up should combine clinical symptoms with imaging studies to screen for long-term complications [6, 7]; for adolescent patients, a balance must be struck between treatment efficacy and organ preservation in order to minimise surgical trauma [8]. The patient in this case made a good recovery following standardised treatment, demonstrating that timely diagnosis, standardised surgery and long-term follow-up are key to improving the prognosis in such complex cases.

## Conclusion

In summary, penetrating anal injury resulting from a fall from a height is a rare form of high-energy trauma, often accompanied by severe multi-organ injury, and presents significant challenges in terms of diagnosis and treatment. In this case, the adolescent patient sustained multisystemic injuries caused by multiple wooden foreign bodies, followed by rare complications that emerged in stages: early rupture of a pseudoaneurysm and delayed formation of a giant bladder stone. The injury pattern, clinical course and rarity of this case render it highly novel.

Rapid initial assessment, diagnostic whole-abdominal CT scanning and thorough emergency surgery are crucial for acute management. Intraoperative bladder exploration and lavage must be comprehensive to rule out submucosal foreign bodies; prior to removal of the urinary catheter, bladder healing must be objectively confirmed via cystography or cystoscopy. Wooden foreign bodies are difficult to visualise and prone to fragmentation; therefore, meticulous exploration and long-term follow-up, including urine analysis and cystoscopy, are essential to prevent late complications such as retained fragments, stone formation and infection.

For adolescent patients, it is essential to adopt a treatment strategy that balances injury control with the preservation of organ function. A staged, individualised treatment plan combining emergency open surgery, minimally invasive interventional radiology, and delayed definitive surgery can significantly improve the prognosis. The experience from this case provides valuable guidance for the early recognition, precise management, and prevention of complications in similar rare traumatic injuries.

## Supplementary Information

Supplementary Material 1.

## Data Availability

No datasets were generated or analysed during the current study.

## References

[1] Stawicki SP, Evans DC, Cipolla J, et al. Retained surgical foreign bodies: a comprehensive review of risks and preventive strategies. Scand J Surg. 2009;98(1):8–17. 10.1177/145749690909800103. 19447736 10.1177/145749690909800103

[2] Klein J, Farman J, Burrell M, Demeter E, Frosina C. The forgotten surgical foreign body. Gastrointest Radiol. 1988;13(2):173–6. 10.1007/BF01889051. 3360252 10.1007/BF01889051

[3] Ponrartana S, Coakley FV, Yeh BM, et al. Accuracy of plain abdominal radiographs in the detection of retained surgical needles in the peritoneal cavity. Ann Surg. 2008;247(1):8–12. 10.1097/SLA.0b013e31812eeca5. 18156915 10.1097/SLA.0b013e31812eeca5

[4] Susmallian S, Folv E, Szyper-Kravitz M. Assessing the diagnostic value of radiographs for retained surgical items: a cautionary analysis. Br J Radiol. 2026;99(1177):150–6. 10.1093/bjr/tqaf241. 41143897 10.1093/bjr/tqaf241

[5] Rajput D, Gupta A, Shasheendran S, et al. Laparoscopic Retrieval of a 13-Year-Old Retained Iatrogenic Metallic Foreign Body from the Pelvis: An Uncommon Case Report. Surg J (N Y). 2023;9(1):e62–6. 10.1055/s-0043-1764124. 36873296 10.1055/s-0043-1764124 PMC9981328

[6] Prihadi JC, Hengky A, Ongga L. Cable Wire as a Nidus for Vesicolithiasis: A Case Report. Res Rep Urol. 2025;17:153–7. 10.2147/RRU.S517497. 40357371 10.2147/RRU.S517497 PMC12068404

[7] Bashir SI, Ali YB, Ali EM, Osman Fadl HA, Abdelmoneim Hamza AH, Fadelallah Eljack MM. Delayed intestinal obstruction from an unintentionally retained surgical gauze in a 24-year old woman two years after caesarean section: a case report. Patient Saf Surg. 2023;17(1):19. 10.1186/s13037-023-00371-y. 37480070 10.1186/s13037-023-00371-y PMC10362721

[8] Verma A, Tran Z, Hadaya J, Williamson CG, Rahimtoola R, Benharash P. Factors Associated With Retained Foreign Bodies Following Major Operations. Am Surg. 2021;87(10):1575–9. 10.1177/00031348211024969. 34132106 10.1177/00031348211024969

