# Supplementary material for: Postoperative foreign body retention following anal injury leading to a giant bladder stone: a case report
Source: BMC Urol. 2026 Jun 2;26:182. doi: 10.1186/s12894-026-02210-5 (PMC13417852; doi:10.1186/s12894-026-02210-5)
Supplement: Supplementary file 1 — Supplementary Material 1. [file 12894_2026_2210_MOESM1_ESM.docx]

**CARE Checklist**

| Item | Domain | Requirement | Completed | Location (Line No.) |
| --- | --- | --- | --- | --- |
| 1 | Title | Identify as case report + key clinical content | ✓ | Line 1 |
| 2 | Keywords | Appropriate indexing keywords | ✓ | Lines 42–43 |
| 3 | Abstract | Uniqueness, patient, diagnosis, intervention, outcome, message | ✓ | Lines 24–41 |
| 4 | Introduction | Background, literature gap, rationale | ✓ | Lines 44–78 |
| 5 | Patient Information | De-identified demographics, chief complaint, history | ✓ | Lines 82–90 |
| 6 | Clinical Findings | Physical exam, imaging, lab, key positives | ✓ | Lines 91–106 |
| 7 | Timeline | Clinical course timeline (illness to follow-up) | ✓ | Lines 151–156; Fig 6 |
| 8 | Diagnostic Assessment | Diagnosis, challenges, tests, differential diagnosis | ✓ | Lines 107–132 |
| 9 | Therapeutic Intervention | Surgery, embolization, cystolithotomy, timing, dosage | ✓ | Lines 113–150 |
| 10 | Follow-up & Outcomes | Long-term follow-up, AEs, recovery | ✓ | Lines 151–156 |
| 11 | Discussion | Literature comparison, limitations, lessons | ✓ | Lines 157–220 |
| 12 | Patient Perspective | Patient’s symptoms and experience | ✓ | Lines 82–90; Line 153 |
| 13 | Consent & Ethics | Ethical approval + written informed consent | ✓ | Lines 245–252 |
